# Next-Generation Pertussis Vaccines Based on the Induction of Protective T Cells in the Respiratory Tract

**DOI:** 10.3390/vaccines8040621

**Published:** 2020-10-21

**Authors:** Caitlín Ní Chasaide, Kingston H.G. Mills

**Affiliations:** School of Biochemistry and Immunology, Trinity College Dublin, 2, D02 PN40 Dublin, Ireland; nichasac@tcd.ie

**Keywords:** *Bordetella pertussis*, pertussis vaccine, T cells, Th1 cells, Th17 cells, memory T cells

## Abstract

Immunization with current acellular pertussis (aP) vaccines protects against severe pertussis, but immunity wanes rapidly after vaccination and these vaccines do not prevent nasal colonization with *Bordetella pertussis*. Studies in mouse and baboon models have demonstrated that Th1 and Th17 responses are integral to protective immunity induced by previous infection with *B. pertussis* and immunization with whole cell pertussis (wP) vaccines. Mucosal Th17 cells, IL-17 and secretory IgA (sIgA) are particularly important in generating sustained sterilizing immunity in the nasal cavity. Current aP vaccines induce potent IgG and Th2-skewed T cell responses but are less effective at generating Th1 and Th17 responses and fail to prime respiratory tissue-resident memory T (T_RM_) cells, that maintain long-term immunity at mucosal sites. In contrast, a live attenuated pertussis vaccine, pertussis outer membrane vesicle (OMV) vaccines or aP vaccines formulated with novel adjuvants do induce cellular immune responses in the respiratory tract, especially when delivered by the intranasal route. An increased understanding of the mechanisms of sustained protective immunity, especially the role of respiratory T_RM_ cells, will facilitate the development of next generation pertussis vaccines that not only protect against pertussis disease, but prevent nasal colonization and transmission of *B. pertussis*.

## 1. Introduction

Pertussis, or whooping cough, is caused by the Gram-negative bacterium *Bordetella pertussis*, which infects the upper and lower respiratory tract, causing considerable morbidity in children and adults and severe disease that can be fatal in infants [1]. The clinical features of disease include a paroxysmal cough, creating a characteristic ‘whoop’, leukocytosis and in some cases pneumonia. Dissemination of *B. pertussis* to other organs occurs very rarely, and only occurs in immunocompromised individuals [2,3]. Whole cell pertussis (wP) vaccines, developed in the 1940s are effective at preventing pertussis, but are associated with significant adverse events, including febrile convulsions [4,5]. This led to their discontinuation in most high-income countries and the development of acellular pertussis (aP) vaccines, now widely used in these countries [6]. aP vaccines contain between 2 and 5 *B. pertussis* antigens, all of which include the major symptom-causing virulence factor, pertussis toxin (PT), as well as various combinations of filamentous hemagglutinin (FHA), pertactin (PRN) and fimbriae 2 and 3 (FIM2/3) [7,8]. aP vaccines administered as part of the primary immunization of infant and young children also contain diphtheria and tetanus toxoids, termed DTaP, whereas booster vaccines, termed Tdap, have reduced antigen content [9,10]. All current aP vaccines are formulated with alum as the adjuvant. Clinical trials carried out in the 1990s showed that the aP vaccines had excellent safety profiles and were capable of preventing severe disease [11,12,13]. However, recent studies in animal models have indicated that immunization with aP vaccines does not prevent nasal colonization or transmission of *B. pertussis* [14,15,16].

Pertussis remains a global problem with the highest case rates of any vaccine-preventable disease [17]. Many pertussis-related deaths still occur in low- and middle-income countries, however, these deaths are not attributable to poor efficacy of wP vaccines but to gaps in vaccination coverage and limited access to healthcare in these countries [18,19]. However, a resurgence in pertussis has been observed in the last decades in countries with high aP vaccine coverage, suggesting that immunization with aP vaccines does not generate sustained sterilizing immunity against *B. pertussis* [20]. Furthermore, epidemiological data have demonstrated that immunization with wP vaccines is more effective at preventing cases of pertussis than aP vaccines [21,22]. Currently, the World Health Organization has recommended that countries still using wP vaccines should not switch to aP vaccines, but it is generally accepted that the reintroduction of wP vaccines would not be possible in countries where it is deemed unsafe.

There are a number of possible reasons for the failure of aP vaccines to generate potent and sustained immunity. In addition to the problem of pertussis in infants too young to be immunized, there are increasing numbers of cases in preadolescents, adolescents and adults, suggesting immunity wanes after immunization with aP vaccines. Indeed, rapid waning of protective immunity has been reported even after five doses of DTaP [23,24]. Although booster vaccination may provide a solution, Tdap boosting only provides significant protection for up to 1 year, and protection substantially wanes 2–3 years later [25]. The emergence of *B. pertussis* strains with deletions or mutations in the antigens present in aP vaccines, which may be caused by vaccine-driven immune selective pressure, could also provide an explanation for the persistence of *B. pertussis* in populations immunized with aP vaccines [26,27,28].

Weak or inappropriate cell-mediated immune responses generated by aP vaccines may also be responsible for the resurgence of pertussis in vaccinated populations. Immunization with aP vaccines induces strong serum immunoglobulin G (IgG) and predominantly Th2-type T cell responses [29]. However, extensive studies in mouse and baboon models of *B. pertussis* infection have demonstrated that robust Th1 and Th17 cell responses are required for optimum protective immunity [30,31,32]. Furthermore, recent reports have indicated that tissue-resident memory T (T_RM_) cells in the upper and lower respiratory tract induced by previous infection or intranasal (i.n.) immunization play a crucial role in long-term protective immunity in the nose and lungs [15,32,33,34].

While immunization with aP vaccines appears to be capable of protecting against severe pertussis disease in infants, there is increasing evidence from animal models that the immunity generated with aP vaccines does not prevent infection of nasal mucosae [14,15]. Asymptomatic carriage in aP-immunized baboons can lead to the transmission of *B. pertussis* to naïve animals [14] and mathematical modelling studies have suggested that asymptomatic carriage and transmission may also occur in the human population and may account for the recent resurgence of pertussis cases in certain countries [35]. This poses a significant risk to infants who have not yet received or completed their DTaP immunization program, and it may impede the potential to generate herd immunity in the population [24,36]. Recent studies have suggested that local immunity, especially IL-17 producing T_RM_ responses, as well as mucosal IgA, may be central to the induction of sterilizing protective immunity in the nasal cavity [33,34]. Therefore, strategies for the design of improved pertussis vaccines must consider the induction of mucosal immunity in the respiratory tract.

This review outlines the state of the art in pertussis vaccine research, discusses current licensed vaccines, vaccines in development, as well as those at an experimental stage, and focuses particularly on how immunity is achieved at a molecular and cellular level. We also discuss the mechanism of natural and vaccine-induced immunity and how effective pertussis vaccines can be designed using immunization approaches that induce cellular as well as humoral immunity, especially local T cell responses in the respiratory tract.

## 2. Immune Responses That Control a Primary Infection with *B. pertussis*

Natural immunity generated by previous infection is considered to be more effective than immunity generated with aP or wP vaccines. Therefore, an understanding of the mechanism of natural immunity in the respiratory tract should assist in the design of a more effective pertussis vaccine. Following recovery from infection with *B. pertussis*, convalescent animals and humans have long-term, but not life-long, protection against subsequent infection through the generation of immunological memory [37]. Although correlates of protective immunity have not yet been fully elucidated, or unanimously agreed upon, there is a wealth of knowledge from recent research on the mechanisms of innate and adaptive immunity to *B. pertussis* that control primary infection and prevent re-infection with *B. pertussis*.

Primary infection with *B. pertussis* can broadly be described in three stages, which include the recognition, bactericidal clearance and memory stages [38]. During the recognition stage, bacteria adhere to ciliated epithelia of the upper respiratory mucosa, and *B. pertussis* is detected by resident innate immune cells, which provide a rapid first line of defense [39,40]. These cells include airway mucosal dendritic cells (DCs), which reside between epithelial cells and survey airway mucosa for pathogens [41]. Alveolar macrophages (AMs), present on the mucosal surface of the lungs, phagocytose and kill bacteria rapidly upon their recognition. Depletion of AM using chlodronate liposomes increases *B. pertussis* burden in the respiratory tract of mice [42]. The recognition of *B. pertussis* pathogen-associated molecular patterns (PAMPs), such as pertussis toxin (PT), lipopolysaccharide (LPS) and lipopeptides, by pattern recognition receptors (PRRs) is crucial for macrophage and DC responses. Mice lacking the toll-like-receptor (TLRs) adaptor, MyD88 adaptor-like protein (Mal), have increased infection in the lungs and bacterial dissemination to the liver, with often fatal consequences [43]. These mice also show reduced lung resident AMs following *B. pertussis* infection, whereas wild-type (WT) mice had increasing numbers of AMs in the lung throughout the course of infection [44]. Airway mucosal DCs process bacterial antigens migrate to the lymph nodes, where they present peptide antigens to naïve T cells [45].

Studies in mouse models have shown that in the bactericidal stage, inflammatory macrophages and DCs infiltrate the lung from the periphery. In addition to their function as antigen presenting cells (APCs), DCs also produce pro-inflammatory cytokines and chemokines in response to PAMPs, including LPS binding to TLR4, that promote the recruitment and activation of other innate immune cells and effector T cells. DCs and macrophages also produce the anti-inflammatory cytokine IL-10, which may protect the lung from excessive inflammation and immunopathology [46]. DCs expressing the integrins CD11c, CD8α and the E-cadherin receptor, CD103, infiltrate the lung early in *B. pertussis* infection and are required for effective bacterial clearance [47]. IL-17-producing γδ T cells are detected in the mouse lung as early as 2 h post-infection [48]. Inhibition of this early innate IL-17 produced by γδ T cells, by treatment of mice with IL-17 neutralizing antibodies 1 day prior and 2 days after infection, led to increased bacterial burden at 3 and 7 days post bacterial challenge and this was associated with reduced local production of protective antimicrobial peptides (AMPs) in the lungs [48].

IL-17 produced by γδ T cells and Th17 cells helps to promote recruitment of neutrophils to the respiratory tract. Neutrophil numbers peak in the lung at 5-7 days post challenge in mice, and have the capacity to phagocytose and kill *B. pertussis*, but are not essential for clearance of bacteria from the lungs during primary infection [49,50]. They do, however, play an important role in the protection of immune mice from reinfection, which is believed to be due to bacterial killing via antibody-mediated phagocytosis [49]. Furthermore, we have found that neutrophils play a central role in clearance of primary and secondary infections of the nasal mucosae with *B. pertussis* (Borkner, Curham and Mills, unpublished data).

NK cells, which infiltrate the lungs of mice around day 7-14 of infection, are an important early source of IFN-γ that activates the bactericidal activity of macrophages [51] and promotes phagolysosomal maturation [52,53]. Macrophages and epithelial cells can act as a niche for intracellular survival of *B. pertussis* and IFN-γ appears to play a key role in eliminating this intracellular reservoir of live bacteria [54]. IFN-γ produced by NK cells also promotes polarization of Th1 cells, and depletion of NK cells causes a shift from Th1 to Th2 responses and leads to bacterial dissemination to the liver [51].

While innate immune responses can effectively limit bacterial growth in the respiratory tract, they do not clear the infection with *B. pertussis*. The adaptive arm of the immune system is required for bactericidal clearance of *B. pertussis*. CD4 T helper (Th) cells are activated by *B. pertussis* antigens presented by APCs in lymph nodes draining the respiratory tract. These CD4 T cells undergo clonal expansion and are indispensable for clearance of *B. pertussis* from the lungs and probably the nasal cavity [52,55]. There is strong evidence that Th1 cells are responsible for the effective clearance of *B. pertussis* from the lungs and there is emerging evidence that Th17 cells mediate clearance from the nasal cavity [16,56]. CD4 T follicular helper (Tfh) cells play an important role in the formation of germinal centers (GCs) and the induction of antibody production and affinity maturation by B cells [57]. Tfh cells are induced by *B. pertussis* infection and may also play a role in generating of *B. pertussis*-specific antibodies (Wilk, Allen and Mills, unpublished data).

Finally, in the **memory** stage, following clearance of *B. pertussis* from the respiratory tract, the number of effector T and B cells contracts, but populations of memory B and T cells are generated and persist to maintain long-lived immunity against re-infection [37,55,56].

## 3. Natural and Vaccine-Induced Immunity-Correlates of Protection

### 3.1. Antibody Responses

#### 3.1.1. Serum IgG

**Figure 1:**
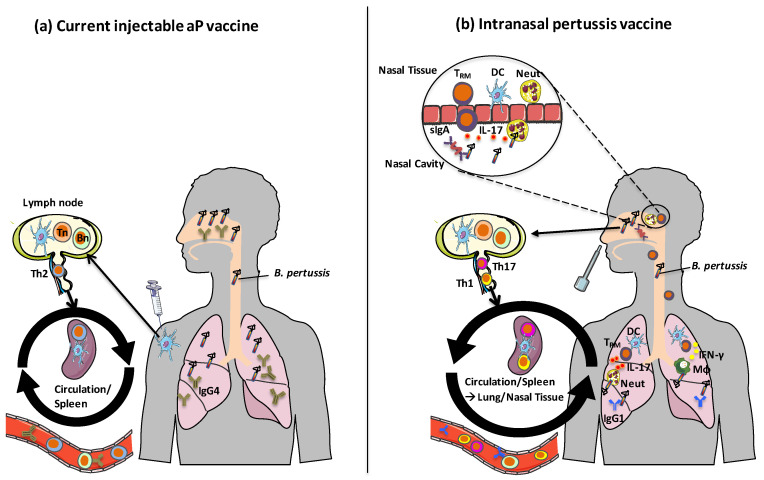
Mechanisms of action of current and future pertussis vaccines. (**a**) Following immunization with current injectable aP vaccines formulated with alum, DCs at the site of injection take up antigens and migrate to draining lymph nodes where they activate differentiation of Th2 cells from naive T cells. Memory Th2 cells circulate, but do not home to respiratory tissues. IgG4 (IgG1 in mice) is produced by antigen-specific B cells, which protects against toxin-mediated pertussis disease but does not protect against nasal colonization with *B. pertussis*. (**b**) Following intranasal immunization with aP vaccines and novel adjuvants, pertussis OMV vaccines or live attenuated pertussis vaccines, local DCs are activated in the respiratory tract. These DCs migrate to draining lymph nodes and activate differentiation of naïve T cells into Th1 and Th17 cells, which have homing properties that facilitate migration to respiratory tissues. Th1 and Th17-type respiratory T_RM_ cells produce IFN-γ and IL-17, which promote recruitment of macrophages and neutrophil to the lungs and nasal mucosae. Intranasal immunization with these vaccines also leads to B cell activation and the production of IgG1 (IgG2a/c in mice) antibodies as well as sIgA. Images from Servier Medical Art (www.smart.servier.com). Tn: naïve T cell, Bn: naïve B cell, T_RM_: tissue-resident memory T cell, DC: Dendritic cell, sIgA: secretory immunoglobulin-A, MΦ: macrophage.

Current aP vaccines generate high levels of antibody and associated Th2 responses (Figure 1), and this is consistent with many alum-adjuvanted vaccines [58,59]. Clinical trials in 1990s demonstrated that immunization of children with aP vaccines induced potent serum IgG responses specific for the vaccine antigens and prevented severe pertussis [13,60]. However, recent evidence suggests that aP-induced immunity does not confer protection against bacterial carriage, which is reflected in an increase in the incidence of pertussis in countries with high vaccine coverage [61,62]. The nature of the antibody isotypes generated, the limited breadth of their antigenic specificity as well as rapid waning of serum antibody concentrations may contribute to this suboptimal protection [23,63].

During natural infection with *B. pertussis*, antigen-specific IgG is only detectable in the serum late in infection when the bacteria are almost cleared from the lung, suggesting that circulating antibodies do not play a major role clearing a primary infection with *B. pertussis*, but may play a role in preventing re-infection [55]. Infection with *B. pertussis* or immunization with wP vaccines induce IgG2a/IgG2c in mice and a corresponding IgG1 response in humans. However, the strong serum antibody response generated by aP vaccines is predominantly of the IgG1 subclass in mice, or, its functional equivalent, IgG4 in humans [64,65]. IgG subclasses are determined by class switching of mature B cells, which occurs through cell–cell interaction between B and T cells, as well as by cytokines secreted by T cells [66]. IgG2a/IgG2c-skewed B cell class switching is associated with Th1-derived IFN-γ [67,68], whereas IgG1 antibodies are associated with Th2 interactions and associated cytokine signaling [69].

In addition to overall levels and specificity, antibody function is an important determinant of effective bacterial clearance, especially opsonizing antibodies which bind *B. pertussis* and promote bacterial uptake and killing by macrophages and neutrophils [54,70]. Phagocytosis can also occur by binding of the complement receptor 3 (CR3), but this mechanism of entry is hijacked by *B. pertussis* to promote intracellular survival [70]. In contrast, uptake via the IgG Fc receptor, FcγRIII, leads to increased *B. pertussis* clearance when compared to CR3-mediated phagocytosis [70]. The IgG subclass can influence opsonization and downstream phagocyte effector function [71]. IgG1 and IgG3, present in serum from subjects vaccinated with the live attenuated pertussis vaccine BPZE1, effectively opsonized *B. pertussis*, whereas IgG2 and IgG4 were the dominant subclass, but opsonizing antibodies were low or absent in the serum from individuals immunized with an aP vaccine [72]. Taken together, these studies show that the ratio of different IgG subclasses generated following vaccination may influence the outcome of infection, as a result of functional differences in the antibodies and their associated T cell responses.

#### 3.1.2. Secretory IgA

During primary infection of mice and humans with *B. pertussis*, secretory IgA (sIgA) is detected in nasopharyngeal secretions, before the detection of IgG [55,73]. IgA can provide early defense against bacterial adherence to epithelial cells [73], can opsonize *B. pertussis* and enhance binding, uptake and killing of bacteria in human polymorphonuclear cells, the latter being mediated by the myeloid-expressed IgA receptor, FcαRI [74]. IL-17 production by Th17 cells has been shown to be important for the generation of sIgA. The induction of Th17 cells by parenteral priming followed by an intranasal (i.n.) boosting with a Group A streptococcal C5a peptidase (ScpA) vaccine correlated with increased local sIgA-producing B cells [75]. This IgA-inducing function may be mediated by Th17-driven expression of the poly-Ig receptor (pIgR), which is involved in sIgA secretion from airway lumen epithelial cells [75,76]. A study using the live-attenuated *B. pertussis* vaccine, BPZE1, demonstrated that IL-17-dependent sIgA is not only important in the primary response to *B. pertussis*, but also in vaccine-induced immunity [34]. Transfer of nasal washes from BPZE1-immunized mice conferred protection against *B. pertussis* infection; however, this protection could not be conferred by transfer of nasal washes from IgA or pIgR deficient mice [34].

### 3.2. CD4 Th Cell Subtypes

#### 3.2.1. Th1 Cells Protect in the Lung

The importance of T cell responses in generating long-lived immunity to many pathogens, including *B. pertussis*, is becoming increasingly evident, and natural infection with *B. pertussis* generates strong Th1 and Th17 responses which mediate effective protection against respiratory infection in animal models [30,55,56]. A similar, albeit weaker Th1/Th17-skewed response and associated protection is generated by immunization with wP vaccines [14,15,16]. Conversely, aP vaccines induce strongly Th2-skewed responses, which are associated with the production of the cytokines IL-4, IL-5 and IL-13 [14,16]. Th1 responses have a well-established role in protective immunity in the lungs induced by natural infection with *B. pertussis* in animal models [30,55]. Th1 cells characteristically produce the pleiotropic inflammatory cytokine, IFN-γ, and its signaling is crucial for protection against lung infection with *B. pertussis* in mice [30,52]. Ablation of IFN-γ signaling, using IFN-γ receptor knockout mice (IFN-γR^-/-^), leads to severe disseminating infection and in some cases fatal disease [30]. Th1 cells also play an essential role in protection conferred with wP vaccines; IFN-γR^-/-^ mice immunized with wP vaccines have 100-1000 fold higher *B. pertussis* load in the lung at days 3, 7 and 10 post challenge, when compared with WT mice immunized with the same vaccine [16,77].

Further evidence supporting the crucial role of Th1 cells in host immunity against *B. pertussis* is provided by the immune evasion mechanisms employed by *B. pertussis* to evade the protective role of IFN-γ [78]. IL-10 is an anti-inflammatory cytokine, which can be produced by regulatory T (Treg) cells as well as macrophages and DCs, and myeloid cell-derived IL-10 can promote the induction of Treg cells [79,80]. IL-10-producing Tr1-type Treg cells are induced in the lung during *B. pertussis* infection driven by innate IL-10 induced by FHA [81] and possibly other *B. pertussis* virulence factors. These *B. pertussis*-specific Tr1 cells suppress protective Th1 cells, highlighting the selective advantage gained by *B. pertussis* in subverting host immunity through skewing the T cell response towards a more Treg phenotype [81]. Although the weight of evidence suggests that CD4 Th cells are the major protective T cells in *B. pertussis* infection, CD8 cytotoxic T cells (CTLs), which also produce IFN-γ, may also play a role, especially against intracellular bacteria [82,83].

There is a bias toward Th2 differentiation in neonates [58,84]. This may reflect high expression of IL-4 and the Th2 transcription factor, GATA3 and hypomethylation of the Th2 locus, which includes *il4*, *il13* and *il5* genes [85,86,87]. Nevertheless, there is evidence that both natural infection and immunization with wP vaccines in infants as young as 2–6 months can generate Th1 responses [29,88]. These studies show that the predilection for Th2 immunity in neonates can be overcome through immunization with wP vaccines, suggesting that these vaccines may include appropriate components that stimulate innate immune responses that direct the induction of Th1 cells. This is an important consideration for the design of novel aP vaccines for administration to neonates.

#### 3.2.2. Th17 Cells Protect Against Nasal Colonization

Th17 cells have more recently been identified as key players in protecting mucosal surfaces against infection with candida and extracellular bacteria [89,90,91]. Many early studies on *B. pertussis* focused primarily on the mechanism of immunity in the lung, but recent studies from mouse and baboon models have shown that IL-17 and Th17 cells in the nasal mucosae may play pivotal roles in natural and vaccine-induced immunity in the nasopharynx [33,56,92].

Th17 cells are polarized by the inflammatory cytokines IL-1β, IL-23 and IL-6. Early evidence for the role of Th17 cells in protection against *B. pertussis* came from studies that showed that immunization with a wP vaccine generated a population of IL-17-producing CD4 T cells [93]. These Th17 cells played a role in wP vaccine-induced protection against lung infection and their induction was dependent on innate TLR4 signaling and the production of IL-23 and IL-1, highlighting the importance of vaccine-induced innate immune activation in promoting effective adaptive immune responses [93]. The generation of *B. pertussis*-specific Th17 cells is dependent on NLRP3 inflammasome-driven IL-1β production in macrophages or DCs and this is activated by the pore-forming capacity of the *B. pertussis* virulence factor, adenylate cyclase toxin (ACT) [94].

Th17 cells are necessary for the effective clearance of *B. pertussis* from the lungs by promoting recruitment of neutrophils and inflammatory macrophages to the respiratory tract [16,93]. Mice defective in IL-17 have impaired clearance of *B. pertussis* from the lung, which is associated with reduced neutrophil recruitment following *B. pertussis* challenge [16,94]. Adoptive transfer of Th1 or Th17 cells from convalescent to naïve mice enhanced clearance of *B. pertussis* from the lung, but the greatest protection was observed when a combination of Th1 and Th17 cells were transferred, indicating a protective role in the lungs for both cell types [16].

While Th1 and Th17 cells contribute to protective immunity generated by immunization with wP vaccines, studies in IL-4^-/-^ mice demonstrated that the strong Th2-skewed cellular responses generated following immunization with aP vaccines were dispensable for the protection in the lungs [16]. However, aP-induced protection in the lungs was significantly impaired in IL-17-defective mice providing further evidence of a role for Th17 in pertussis vaccine-induced immunity [16].

In the baboon model, sterilizing immunity is generated by previous infection with *B. pertussis* and this is associated with production of mucosal IL-17 and the generation of long-lived memory Th17 cells [56]. High concentrations of IL-17 were found in nasal washes on days 5 and 7 of *B. pertussis* infection and this was accompanied by high levels of Th17-polarising cytokines IL-1β, IL-23, IL-6 [56]. Long-lived CD95^+^ CD4^+^ memory T cells that secreted IL-17 or IFN-γ were detected in baboons up to two years following challenge with *B. pertussis* [56]. Furthermore, immunization with wP vaccines protected against nasopharyngeal colonization and disease [14]. However, while natural infection or immunization with a wP vaccine generated Th1/Th17 responses, aP vaccines generated predominantly Th2 responses [14]. Furthermore, immunization of baboons with an aP vaccine protected against clinical disease, but was ineffective at protecting against nasal colonization with *B. pertussis* or transmission of bacteria to naive baboons [14]. While natural infection or immunization with either vaccine generates strong IgG responses against PT, FHA, PRN and FIM, the inability of aP vaccines to prevent nasal infection may be attributed to their limited ability to induce an effective cellular immune response, especially memory Th17 cells in the respiratory tract. 

In humans, vaccination with wP vaccines has also been shown to generate Th17 as well as Th1 responses [95]. The nature of the initial priming dose of the vaccine dictates long term polarization of memory T cells [59,95,96]. A recent study reported that boosting with Tdap more than 15 years after the primary vaccination with five doses of DTwP generated greater PT-specific CD4 T cell responses, enhanced proliferative capacity of memory T cells and induced higher numbers of Th17 and Th1 cells than in individuals primed with DTaP vaccines [95]. Transcriptomic analysis showed upregulation of IL-17 and IFN-γ gene expression in the DTwP-primed individuals, and upregulation of IL-4 and IL-9 in the DTaP-primed individuals [95]. A study in children primed with aP or wP vaccines and boosted with an aP vaccine at 9 years of age revealed lower *B. pertussis*-specific cellular immune responses and reduced Th1/Th2 ratio in the aP-primed individuals [96]. Together, these studies indicate that the priming vaccine is of utmost importance in determining enduring T cell differentiation and memory responses.

### 3.3. T_RM_ Cells Maintain Sustained Protective Immunity in the Respiratory Tract

Circulating memory T cells have classically been subdivided into effector memory (T_EM_) and central memory (T_CM_) cells based on both their homing and effector function [97]. More recently, a distinct subset of memory T cells, tissue-resident memory T (T_RM_) cells have been identified, which remain in tissues following infection, with little or no recirculation [98,99,100]. CD4 and CD8 T_RM_ cells in humans and mice express the transmembrane C-type lectin, CD69, which plays a role in their retention within peripheral tissues [101,102,103]. CD8 and some CD4 T_RM_ cells also express the α_E_ integrin CD103. CD4 T_RM_ cells that co-express CD103 and CD69 are found in the lungs of mice following *B. pertussis* infection [32].

T_RM_ cells maintain immunological memory at mucosal surfaces and are poised to act rapidly as first responders at mucosal sites [104], recruiting innate and adaptive immune cells to the site of infection [105,106]. T_RM_ cells possess innate-like properties and can be activated by cytokines, in the absence of TCR ligation with a cognate antigen [107]. CD8 T_RM_ cells are particularly important in viral infections and have been studied in the greatest detail [99,108,109]. However, CD4 T_RM_ cells also play a role in protection against viral infections at mucosal sites [103,110]. CD4 T_RM_ cells are retained in the lungs of mice following influenza challenge and traffic back to the lungs of naïve mice following adoptive transfer [103].

There is growing evidence that local memory T cells in the respiratory tract may mediate protective immunity against certain pathogens independent of antibody responses. Natural infection to respiratory syncytial virus (RSV) generates both lung and airway CD8 T_RM_ cells, which protect in the absence of antibodies [111]. CD4 T_RM_ cells induced in the airways following intranasal immunization with Venezuelan equine encephalitis replicon particles expressing the SARS-CoV nucleocapsid protein produced IFN-γ that mobilized CD8 T cells and protected against SARS-CoV challenge in mice [112]. Respiratory CD4 and CD8 T_RM_, which are induced by the infection of mice with influenza virus or through i.n. immunization with a live-attenuated virus, mediate protection against challenge with heterosubtypic influenza strains in mice [113,114]. These T_RM_ cells have been shown to confer protection independently of antibody responses [113,115]. IL-17-producing CD4 T_RM_ cells expanded in the lungs following *Streptococcus pneumoniae* infection were capable of preventing reinfection with a heterotypic pneumococcus [116]. Interestingly, virus-specific, lung CD8 T_RM_ cells have been shown to be protective against bacterial pneumonia challenge through bystander activation [117]. Lung resident CD4 T_RM_, cells induced by various tuberculosis (TB) vaccines mediate protection against *M. tuberculosis* challenge in animal models [118,119,120].

The recent understanding that *B. pertussis* can asymptomatically colonize the nasal cavity has focused attention on the role of local immunity in the respiratory tract. CD4 T_RM_ cells are expanded in the lung and nasal tissue during infection of mice with *B. pertussis* [15,32]. Blocking accumulation of T_RM_ cells in the lungs during primary infection with *B. pertussis*, using the sphingosine-1-phosphate (S1P) receptor agonist, FTY720, which blocks T cell egress from lymph nodes, enhanced the bacterial burden in the lungs [32]. However, when mice were treated with FTY720 prior to and during secondary infection, the bacterial burden was similar to untreated convalescent mice, indicating that T_RM_ cells are retained in the lung tissue after the clearance of *B. pertussis*, and proliferate locally in response to re-infection. These protective CD4 T_RM_ cells secreted IL-17 or both IL-17 and IFN-γ, and expressed CD69, with or without CD103 [32].

IL-17 and IFN-γ secreting CD4 T_RM_ cells accumulate in the lung parenchyma and nasal tissue soon after *B. pertussis* challenge of mice immunized with a wP vaccine [15]. Mice immunized with a wP vaccine rapidly cleared bacteria from the lungs following *B. pertussis* challenge. In contrast, bacterial clearance from the lungs was delayed in mice immunized with an aP vaccine. However, a striking difference was observed in the nasal cavity. Mice immunized with aP vaccines had similar or greater bacterial burden in the nasal mucosae than naïve mice following challenge with *B. pertussis*. In contrast, mice immunized with a wP vaccine had significantly reduced bacterial counts by day 3 post challenge and cleared the infection completely by day 14. CD4 T_RM_ cells transferred from the lungs of convalescent to naïve mice one day before *B. pertussis* challenge migrated to the lungs and conferred significant protection against challenge. Conversely, transfer of splenic memory T cell from previously infected, aP or wP-immunized mice did not lead to an accumulation of T_RM_ cells in the lung_,_ or confer any protection in recipient mice [15].

*B. pertussis*-specific CD69^+^ CD4 T_RM_ cells were found to persist in the mouse lungs for at least 7 months after *B. pertussis* challenge. Sustained protective immunity in the nasal cavity, generated by natural infection or immunization with a wP vaccine, was strongly correlated with IFN-γ-producing and, to an even greater extent, IL-17-producing CD69^+^ CD4 T_RM_ cells [15]. Therefore, there is growing evidence to suggest that the induction of respiratory T_RM_ cells may be central to the generation of sterilizing immunity and long-term protection against *B. pertussis* infection of the lung and nasal mucosae.

## 4. Experimental Vaccines

It is now evident from studies in mice and baboons that immunization with aP vaccines is ineffective at generating protective immunity against nasal colonization with *B. pertussis* [17] (Figure 1). Although wP vaccines do confer some protection against nasal colonization, they are not as effective as previous infection with *B. pertussis* [14,15] and are associated with significant side effects. Therefore, new pertussis vaccines are in development based on live attenuated *B. pertussis*, pertussis outer membrane vesicles (OMVs) or aP vaccines formulated with novel adjuvants.

### 4.1. Live Attenuated Pertussis Vaccine-BPZE1

Locht and colleagues developed a live attenuated *B. pertussis* from the Tohama-1 strain, termed BPZE1, in which three virulence factors, PT, dermonecrotic factor (DNT) and tracheal cytotoxin (TCT) are genetically modified or removed [121]. Studies in mice showed that a single i.n. immunization with BPZE1 effectively colonized the respiratory tract without generating pathology in the lung and conferred superior protection against infection with virulent *B. pertussis* compared with two doses of an aP vaccine [121]. BPZE1 has also been shown to be safe in humans in phase 1 clinical trials [122]. Furthermore, studies in baboons showed that a single i.n. dose of BPZE1 protected against nasopharyngeal colonization and pertussis disease [14,92].

BPZE1 also generates long-lived protective immunity to *B. pertussis* in mice and this has been attributed to antibody and Th1/Th17 responses [123,124,125]. Transfer of serum antibodies or splenic CD4 T cells from BPZE1-immunized mice provided protection against infection in the lung with virulent *B. pertussis*, but did not protect in the nasal cavity [34,124]. However, transfer of nasal washes from BPZE1-immunized mice to naïve mice did protect against nasal colonization [34]. BPZE1-induced protection against nasopharyngeal colonization was dependent on the production of local sIgA and IL-17. BPZE1 immunization also generated nasal CD4 T_RM_ cells, which produced IL-17 and, to a lesser extent, IFN-γ, and protection was greatly diminished in IL-17^-/-^ mice [34]. This study also showed that long-term protection was maintained in the nasal cavity for up to 10 months post immunization. However, nasal washes transferred at the later time point did not protect naïve mice, suggesting that sustained protective immunity generated by BPZE1 in the nasal cavity may be due to local IL-17-producing T_RM_ cells [34].

It was also reported that BPZE1 could induce rapid protection against *B. pertussis* infection in mice soon after immunization [126]. Mice were significantly protected at 1 week after immunization and this was dependent on TLR4 and the TLR adaptor protein, MyD88, and was completely independent of adaptive immune responses [126]. These findings suggest that BPZE1 is effective at stimulating protective innate immune responses. The studies in mouse models were translated to humans through the demonstration that human monocyte-derived DCs, cultured in vitro with BPZE1, mature similarly to those cultured with virulent *B. pertussis* and, when co-cultured with naïve T cells, these DCs promoted Th1 and Th17 differentiation [127]. A vaccine study in humans showed that immunization with BPZE1 induced strong Th1 responses, with IgG1/IgG3 serum antibodies, whereas volunteers immunized with an aP vaccine had Th2-biased responses [72]. Serum antibodies from the BPZE1-immunized individuals promoted neutrophil reactive oxygen species (ROS) production and had bactericidal killing capacity [72]. Systemic Th17 responses were not detected in this study, however mucosal T cell responses were not assessed, thus, it remains to be seen whether local airway Th17 cells are generated by immunization with BPZE1 in humans [72]. BPZE1 is currently being tested in Phase 2 clinical trials as a single i.n. immunization (NCT03541499) or comparison with aP vaccine in various prime-boost vaccination strategies (NCT03942406).

### 4.2. OMV Vaccines

OMVs are spherical structures derived from the cellular envelope of Gram-negative bacteria, which bud from actively growing cells and are composed of their outer membrane containing periplasmic components [128]. An advantage of pertussis OMV over aP vaccines is that they contain a wider variety of bacterial antigens in their natural conformation, which can elicit a broader immune response, without the same risk of over-exuberant inflammatory responses observed with wP vaccines [129,130]. Studies in mice suggest that pertussis OMV vaccines delivered parenterally generate mixed Th1/Th2/Th17 responses as well as CD4 T_RM_ cells, conferring protection greater than that induced with aP vaccines, and similar to that induced with wP vaccines, whilst displaying a better safety profile [129,131,132]. Zurita et al. showed that immunization of mice with a pertussis OMV vaccine conferred greater protection than an aP vaccine against a PRN(−) strain of *B. pertussis*. Immunization with the pertussis OMV vaccine also induced *B. pertussis*-specific splenic Th1 and Th17 memory responses, as well as IL-17- and IFN-γ-producing CD4 T_RM_ cells in the lungs [131]. Long-term protection conferred by OMV vaccines may be mediated by cytokine-secreting T_RM_ cells in the lung [131,133].

An OMV vaccine produced from the *B. pertussis* Tohama-1 strain, which expresses the *B. bronchoseptica* gene *pagL* encoding lipid A 3-deacylase PagL (OMV_BpPagL_), enhanced protection in the lungs induced with a Tdap vaccine, and provided long-term protection against multiple *B. pertussis* strains at 9 months post-immunization [133]. Studies in mice suggested that the OMV_BpPagL_ vaccine was safer than WT OMVs; mice immunized i.n. with OMV_BpPagL_ had less weight loss and reduced IL-6 and IL-1 production compared with mice immunized with WT OMVs [134]. OMVs generated from the *B. pertussis* B1917 strain provided comparable protection to a wP vaccine in mice, but induced lower serum IL-1β and IL-6 concentrations. Induction of IL-1β in particular has been associated with wP-induced neurological effects [135].

Immunization with pertussis OMV vaccine generates antibody responses with a higher IgG2a/IgG1 ratio and stronger opsonizing activity when compared with antibodies induced with aP vaccines [132]. The breadth of the antibody responses is also greater after immunization with pertussis OMV vaccines, with antibodies specific for both aP and non-aP antigens, such as GroEL, Vag8 and BrkA [44,132].

Pulmonary administration was found to be an effective route for the delivery of a pertussis OMV vaccine and induced Th17 and Th1 responses locally in the lung and systemically, as well as antigen-specific IgA production [136]. However, pulmonary immunization did not prevent nasal colonization with *B. pertussis* [136]. In contrast, immunization with OMV by the i.n. but not the s.c. route conferred significant protection in the nasal cavity [137]. Protection was associated with the induction of mucosal IL-17 and IFN-γ, increased lung and nasal IgA as well as strong systemic Th17 responses [137].

### 4.3. aP Vaccines with New Antigens

One explanation for the failure of aP vaccines to confer long lasting protection in immunized individuals, is the limited number of antigens, which results in a narrower range of *B. pertussis*-specific immune responses [72]. In addition, there is evidence that aP vaccines with a limited number of antigens may have facilitated immune selective pressure that has led to the emergence of PRN(−) strains of *B. pertussis* [26,138]. PRN(−) strains have been shown to outcompete PRN(+) strains in aP-immunized mice [28]. Strains with mutations in the promoter region of the *ptx* gene which encodes PT have also been identified [139]. *B. pertussis* strains lacking both PRN and PT are rarer, but have also been documented, as have reports of FHA and FIM2/3 deficient strains [140,141,142,143]. Therefore, addition of new antigens to aP vaccines may increase protection and reduce the effects of immune selective pressure. 

FIM2/3 are currently included in five-component aP vaccines, but not in two- and three-component vaccines, and clinical trials have shown that aP vaccines which contain FIM2/3 may be more efficacious in preventing pertussis disease [12,13]. Studies in mice showed that the addition of high doses of FIM2/3 to commercial, two-, three- and five-component aP vaccines increased protection in the lungs following challenge with a Prn(−) strain of *B. pertussis*.

*B. pertussis* virulence is regulated by the BvgAS system, which includes two components, the histidine kinase, BvgS, and the response regulator BvgA [144]. The BvgAS system positively regulates almost all *B. pertussis* virulence factors, including current aP antigens PT, FHA, PRN and FIM2/3 [145]. Adenylate cyclase toxin (ACT) is encoded by the *cyaA* gene, activated by BvgA [146], and plays a central role in inhibiting the host immune response to *B. pertussis* and promotes bacterial survival [147]. Although ACT is an important *B. pertussis* virulence factor, it is not currently included in any commercial aP vaccine. Early evidence that ACT may be an immunodominant antigen came from studies showing that *B. pertussis*-infected individuals had high levels of anti-ACT antibodies [148]. Anti-ACT antibodies are also generated following immunization with wP vaccines and are present in newborn cord blood [149,150]. Native and recombinant ACT have been shown to have protective function in animal models [151,152,153,154]. Addition of active or enzymatically inactive ACT to aP vaccines promoted a shift from Th2 to a mixed Th1/Th2 response and enhanced protection against lung infection with *B. pertussis* in mice, however, immunization with ACT alone did not confer protection [155]. ACT in its native form, undergoes quick proteolytic degradation within the cell, complicating its potential use in aP vaccines [156,157]. However, RTX antigens, which are more stable than full-length ACT generate strong ACT neutralizing antibody responses [158]. Addition of C-terminal repeats-in-toxin domain (RTX) of ACT to a low dose (1/80 of human dose) of an aP vaccine enhanced its protective efficacy against lung infection with *B. pertussis* in mice [159].

Bordetella resistance to killing A (BrkA), a virulence factor activated by phosphorylated BvgA, which has autotransporter function and plays a role in bacterial adherence to lung epithelial cells, as well as in evading complement-mediated phagocytosis [160] is another candidate antigen that may improve the efficacy of aP vaccines. Immunization of mice with BrkA alone by the s.c. route did not confer protection against lung infection in mice, but it did enhance protection when added to an aP vaccine comprising FHA and PT antigens [161]. However, another study showed that when compared with control mice immunized with alum only, mice immunization with an alum-adjuvanted mono-component BrkA vaccine by the i.p. route had lower numbers of bacteria in the lungs 7 days post *B. pertussis* challenge [162].

Immunization by the s.c. route with either recombinant BvgA-activated autotransporter, Vag8 or SphB1, adjuvanted with alum conferred some protection against lung infection with *B. pertussis* B1917. However, vaccines containing these antigens did not confer protection in the upper respiratory tract [163]. Although the addition of new antigens may indeed enhance protection in the lung, it is evident that generating protection in the upper respiratory tract will prove more challenging and may not be achievable solely through the addition of antigens to parenterally administered aP vaccines.

### 4.4. aP Vaccines with New Adjuvants

**Figure 2:**
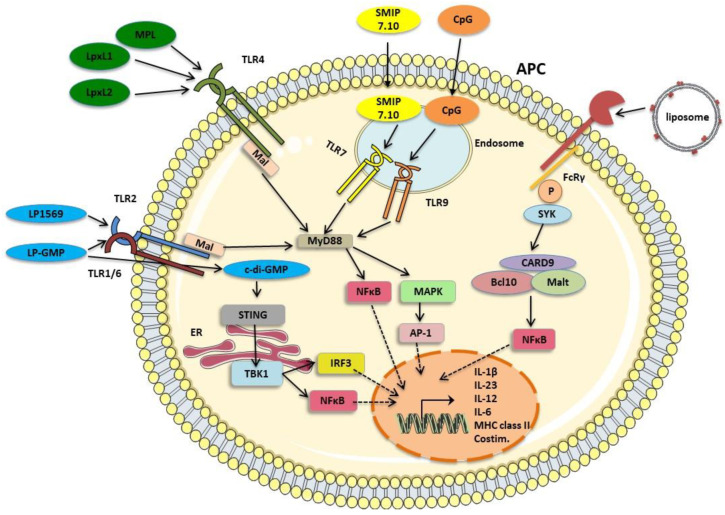
Mechanisms of action of novel adjuvants under investigation in experimental aP vaccines. Activation of signaling pathways in APC by various adjuvants can be harnessed to generate Th1/Th17 polarizing conditions following immunization. These include the TLR2 agonist LP1569 and the TLR4 agonists MPL, LpxL1 and LpxL2 which are synthetic analogues of LPS. SMIP7.10 and CpG ligate the endosomal TLRs, TLR7 and TLR 9 respectively. TLR 2, 4, 7 and 9 signal through the adaptor molecule MyD88, with TLR2 and TLR4 requiring the bridging adaptor Mal to activate MyD88-mediated signaling. MyD88 signaling results in downstream activation of NFκB and MAPKs. MAPKs activate transcription factors such as AP-1. NFκB and AP-1 translocate to the nucleus leading to the transcription of pro-inflammatory and T cell-polarizing cytokines, chemokines, MHC class II and costimulatory molecules. The CLR, Mincle, is ligated by the TDB component (shown in red) of liposomes, which signals via SYK, and subsequently activates CARD9-Bcl10-Malt signalosome and NFκB. c-di-GMP activates the intracellular DNA sensor, STING, located at the ER, and complexed with TBK1 translocates to perinuclear regions, activating NFκB and IRF3. LP-GMP, which combines LP1569 and c-di-GMP adjuvants, signals through TLR2 and STING respectively, resulting in synergistic induction of Th1/Th17 responses. Block arrows denote activation and dashed arrows denote translocation of transcription factors to the nucleus. Images from Servier Medical Art (www.smart.servier.com). APC: antigen presenting cell, TLR: Toll-like receptor, Th: T helper cell, MPL: Monophosphoryl lipid A, LPS: Lipopolysaccharide, MyD88: Myeloid differentiation primary response gene 88, Mal: MyD88 adaptor-like, NF-κB: Nuclear Factor kappa B, MAPK: Mitogen-activated tyrosine kinase, TDB: α,α trehalose 6,6’-dibehenate, SYK: Spleen tyrosine kinase, CARD9: Caspase recruitment domain family member 9, Bcl10: B-cell lymphoma 10, Malt1: Mucosa-associated lymphoid tissue 1, AP1: Activator protein 1, IRF3: Interferon response factor 3, STING: Stimulator of interferon genes, TBK1: TANK-binding kinase 1, MHC Class II: Major histocompatibility complex class II, Costim: costimulatory molecules.

Adjuvants function by activating innate immune cells, which in turn direct adaptive immune responses to antigenic components in a vaccine, and this not only increases the quantity of the immune response but can improve its quality [164]. Natural components of many live attenuated and killed whole-cell vaccines have adjuvant properties, but this can result in excessive unwanted inflammation, as observed in some instances with wP vaccines [4,165]. Subunit vaccines, on the other hand, are often poorly immunogenic without the addition of an appropriate adjuvant [166]. Live *B. pertussis* and wP vaccines contain LPS, which activates DCs through TLR4, and although effective in eliciting strong Th1 and Th17 responses, LPS is not a safe adjuvant for human use [93,167]. However, there are a number of alternative adjuvants based on PAMPs, including TLR agonists, and particulate delivery systems that are safe for use in humans (Figure 2).

#### 4.4.1. Alum

All licensed aP vaccines utilize alum as the adjuvant. Alum has been in common use since the 1920s, when it was first shown to enhance immunogenicity of diphtheria toxoid [168]. Alum or aluminum salts are safe and non-toxic adjuvants, and promote strong antibody and Th2 response, but also induce IL-10 production by DCs and macrophages, which can inhibit polarization of Th1 cells [169] Its beneficial role was initially believed to be due to depot formation, where antigens are sequestered within an alum aggregate at the site of injection, allowing slow release of antigens [170,171]. Furthermore, the particulate antigens generated with alum can be phagocytosed more readily by APCs [172]. Alum can also activate the NLRP3 inflammasome, leading to IL-1β processing [173,174] and this can promote the generation of Th17 responses in mice immunized with aP vaccines [16]. Other studies have shown that NLRP3 may be dispensable for its activity as an adjuvant, suggesting multiple mechanisms of immune activation [175].

Alum can inhibit IL-12 production by DCs thereby generating Th2-skewed CD4 T cell responses [176]. Th2 polarization along with strong antibody responses are effective for vaccines against certain infectious diseases, where their protection clearly correlates with humoral immunity [177]. However, this may not be the case for pertussis, where there is a clear protective role for T cells. An alternative strategy for inducing protective cellular immune responses and memory is through the addition of novel adjuvants to aP vaccines, either replacing alum completely, or adding them to vaccines already formulated with alum.

#### 4.4.2. TLR4 Agonists

The TLR4 agonist LPS is a natural component of the outer membrane of *B. pertussis* and is present in wP vaccines, functioning as a natural adjuvant, which contributes to protective immunity induced with this vaccine [93,178]. TLR4 signaling is crucial for early innate responses and airway neutrophil recruitment during *B. pertussis* infection, and is essential for protection generated with the BPZE1 vaccine [126,179]. LPS is a potent activator of TLR4-dependent pro-inflammatory responses, but because of its toxicity is not safe for use as an adjuvant for human subunit vaccines [180]. To overcome toxicity issue while retaining its adjuvant capacity, LPS derivatives, such as monophosphoryl lipid-A (MPL), have been developed which can bind TLR4, and induce downstream signaling without the risk of adverse events [181]. MPL is an approved adjuvant in vaccines against hepatitis B virus and human papilloma virus [182,183]. Mice immunized with an aP vaccine formulated with MPL had lower bacterial load in the lung post *B. pertussis* challenge, compared to mice immunized with alum-adjuvanted aP [184]. These mice also had higher anti-PT IgG in serum, and reduced *B. pertussis*-specific Th2 responses [184].

An alternative LPS derivative from *Neisseria meningitis*, LpxL2, promoted protective immunity comparable to MPL. The aP vaccines formulated with MPL or LpxL2 induced less IL-6 production in mice than a wP vaccine, suggesting that they may promote protective inflammatory response with a reduced risk of immunopathology [184]. The LPS derivative, LpxL1, also derived from *N. meningitis*, has been shown to shift CD4 T cell responses from a Th2 to Th1/Th17 profile in mice when added to a commercial alum-adjuvanted aP vaccine [185]. Using MHC class II PRN tetramers, the authors showed that the addition of LpxL1 to aP vaccine led to increased PRN-specific CD4 T cells and a greater number of T cells with a T_CM_ phenotype in the draining lymph nodes and blood of immunized mice [185]. Transcriptomic analysis revealed that genes involved in Th2 and Treg polarization were upregulated in mice immunized with the aP vaccine alone, whereas the addition of LpxL1 to an aP vaccine led to an increase in *il17* and *ifnγ* mRNA expression, as well as increasing expression of Th1 and Th17 transcription factor genes, *tbx21* and *rorc*. This demonstrates how the addition of a TLR4 agonist can alter immune responses at a gene expression level [186].

#### 4.4.3. TLR 9 Agonists

Unmethylated single-stranded CpG DNA motifs, which are released from bacteria during infection, activate TLR9 signaling [187]. Synthetic CpG oligodeoxynucleotides (ODNs) that mimic this process have adjuvant capacity, generate strong Th1 responses in mice and humans and are capable of overriding the alum-driven Th2 bias when used in combination with alum [188,189,190]. The addition of CpG ODNs to an aP vaccine, either with or without alum, increased anti-PT IgG2a serum antibody titers [191] and robustly induce IFN-γ-producing splenic T cells [192]. Ross et al. showed that an experimental aP vaccine formulated with CpG alone induced *B. pertussis*-specific Th1 and Th17 responses, and IgG2 antibody responses in mice [16]. This was associated with significant protection against lung infection with *B. pertussis*, with full clearance of bacteria by day 10 post challenge, compared with day 15 for mice immunized with an aP vaccine formulated with alum [16].

A study by Asokanathan and colleagues demonstrated that addition of CpG to an alum-adjuvanted aP vaccine enhanced protection against lung infection of mice with *B. pertussis.* This was associated with increased IFN-γ production in the spleen and increased nitric oxide production in peritoneal macrophages stimulated in vitro with heat-killed *B. pertussis* [190]. However, in this study, substituting CpG for alum did not enhance protection against lung infection.

#### 4.4.4. TLR7 Agonists

TLR7 agonists have adjuvant activity, enhance vaccine efficacy and generate Th1/Th17 responses in a number of infectious diseases [193,194,195]. The adsorption of small molecule TLR7 agonists to alum increases their safety for use as adjuvants, due to reduced systemic inflammation and attenuated cytokine release into the blood, while also improving adjuvant function [196]. Addition of the synthetic TLR7 agonist, SMIP-7.10 to an alum-absorbed aP vaccine significantly enhanced its efficacy and conferred protection against lung infection of mice with *B. pertussis*, comparable to that generated with a wP vaccine [197]. The addition of SMIP-7.10 resulted in a shift from a Th2 response induced with the alum-adjuvanted vaccine to the induction of Th1 and Th17 responses. This was accompanied by an increase in IgG2a and IgG2c antibodies as well as enhanced functional activity, including antibodies that neutralized PT and inhibited FHA binding to human lung epithelial cells [197]. These findings suggest that it may be possible to enhance the efficacy of existing alum-adjuvanted aP vaccines by addition of small molecule TLR agonists that promote protective T cell responses.

#### 4.4.5. TLR2 Agonists

*B. pertussis* expresses a number of lipoproteins that are TLR2 agonists and also have antigenic properties [198]. BP1569 and a synthetic lipopeptide derivative LP1569 have immunomodulatory activity, activating innate immune responses and acted as adjuvants for an experimental aP vaccine in mice. LP1569 induced the production of T cell polarising cytokines, IL-1β, IL-12, IL-23 and enhanced expression of costimulatory molecules, CD80 and CD86, and MHC class II on murine and human DCs [198]. Immunization of mice with an aP vaccine formulated with LP1569 enhanced protection against lung and trachea infection compared with an alum-adjuvanted aP vaccine [198]. Furthermore, strong FHA-specific Th1 and Th17 responses were observed in spleen of mice immunized with the aP vaccine formulated with LP1569, whereas strong Th2 responses were observed in mice immunized with *B. pertussis* antigens alone or with alum [198].

#### 4.4.6. STING and TLR Agonists

Stimulator of interferon genes (STING), which binds cyclic dinucleotides (CDNs), functions as an adaptor molecule for the cytosolic DNA receptor cyclic-GMP-AMP synthase (cGAS), which converts double-stranded (ds)DNA to the second messenger, cyclic GMP-AMP (cGAMP) [199]. STING acts as a PRR and detects bacterial second messengers, such as cyclic-di-GMP (c-di-GMP) and c-di-AMP (c-di-AMP) [200,201]. Ligation of STING by CDNs activates signaling pathways leading to the activation of the transcription factors NF-κB and IRF3 and type 1 interferon gene expression [202,203].

Administration of c-di-GMP to mice by the i.n. route 24 h before *B. pertussis* challenge enhanced bacterial clearance from the lungs of mice [204]. C-di-GMP treatment increased pulmonary neutrophil and macrophage recruitment and induced early macrophage inflammatory protein-2 (MIP-2), which is important for neutrophil recruitment, and macrophage chemotactic protein 1 (MCP-1), which recruits a range of lymphoid and myeloid cells [204,205,206,207]. DC activation and Th1 cytokine production was also enhanced before and after *B. pertussis* challenge in mice treated with c-di-GMP [204]. These findings demonstrated the central role of innate immune stimulation in generating a protective immunity against *B. pertussis* and suggested that c-di-GMP could be a viable adjuvant for a pertussis vaccine.

The adjuvant activity of STING agonists is enhanced when combined with TLR agonists. The combination of agonists for STING and TLR2 or TLR9 induced IL-12 and IL-23 production by innate immune cells, which promoted induction of Th1 and Th17 cells [33,208]. The STING agonist c-di-GMP combined with the TLR2 agonist LP1569, termed LP-GMP, has potent adjuvant activity and when added to an experimental 3-component aP vaccine, containing FHA, PT and PRN, promoted protective immunity against *B. pertussis* in the lower and upper respiratory tract of mice [33]. The most dramatic adjuvant effect was observed when LP-GMP-adjuvanted aP was administered by the i.n. route; this combination conferred sustained protection against nasal colonization and lung infection of mice when challenged with *B. pertussis* up to 10 months after immunization [33]. In contrast, the same aP vaccine formulated with alum did not confer any protection against nasal colonization [14,15,33]. The aP vaccine formulated with LP-GMP induced potent Th17 responses, especially when delivered by the i.n. route. However, parenteral immunization generated stronger systemic *B. pertussis*-specific IFN-γ, indicating stronger Th1 responses [33]. Immunization with LP-GMP-formulated aP vaccine also promoted accumulation of CD69^+^ CD103^+/-^ CD4 T_RM_ cells in the lung and nasal tissue; induction of respiratory T_RM_ cells was most effective following i.n. administration of the vaccine. These respiratory T_RM_ cells predominantly produced IL-17 and the number of these cells strongly correlated with long-term protective immunity in the nasal cavity [33]. This highlights the potential power of combining adjuvants to generate a robust Th1/Th17 response and T_RM_ cells, and demonstrates how mucosal immunization can further enhance protective immunity in the respiratory tract.

#### 4.4.7. Particulate Antigen Delivery Systems, Live Vectors and Nucleic Acid Vaccines

Vaccine delivery vehicles have also been used as tools to improve the efficacy of pertussis vaccines. Microparticles made from the biodegradable polymer poly(lactide-co-glycolide) (PLG) have been utilized as a delivery vehicle for aP vaccines. A single parenteral dose of PLG microparticle-encapsulated FHA and detoxified PT was shown to induce robust Th1 responses and to protect against *B. pertussis* infection in the lungs of mice [209]. Liposomes also have potential as particulate delivery systems for aP vaccines, having already shown promise in experimental vaccines against other infectious diseases, notably *Mycobacterium tuberculosis* [210]. Furthermore, recombinant vector vaccines and mRNA and DNA vaccines are in development for viruses such as SARS-CoV-2 [211] and have potential in the development of new vaccines against *B. pertussis*.

## 5. Route of Vaccine Administration—A Case for an Intranasal Pertussis Vaccine

Most vaccines approved for use in humans are delivered via parenteral routes, mostly intramuscular (i.m.), but also s.c. or intradermal (i.d.) [212]. It is now evident that pathogens that infect mucosal surfaces require effective local immunity and immune memory at the mucosal site of infection. Recent studies in mouse models have demonstrated that the induction of mucosal T_RM_ cells is key to sustained protective immunity against many mucosal pathogens, and immunization at mucosal site of infection appears to be the most effective strategy to achieve this [213]. Currently, the only approved mucosal vaccines are a live attenuated influenza vaccine delivered by nasal spray and a number of oral vaccines against enteric pathogens [214].

Studies that examined the route of administration of TB vaccines have demonstrated that efficacy is often enhanced when a vaccine is delivered by a mucosal route [215,216,217,218,219]. Protection against *M. tuberculosis* conferred by the attenuated *M. bovis* vaccine BCG is more effective following i.n. or intratracheal (i.t.) delivery compared with parenteral administration [220,221,222]. Mucosal immunization of mice with BCG induces a higher frequency of CD4 T_RM_ cells in the lungs when compared with immunization by a parenteral route, and this is associated with enhanced protection against infection in the lungs [215,216].

In the context of *B. pertussis* infection, it is now established that generating strong mucosal responses in the lung and nasal cavity, including the generation of respiratory T_RM_ cells, local IL-17 and sIgA are important for sustained protection against lung infection and nasal colonization [14,32,33,34,131]. However, parenterally delivered alum-adjuvanted aP vaccines do not generate local immunity in the respiratory tract, but instead rely on circulating antibodies driven by systemic T cell responses to prevent severe disease mediated by the *B. pertussis* toxins [14,15,29,223]. Delivery of pertussis vaccines by the i.n. route has the potential to enhance local immune responses in the nasopharynx, and may provide a solution to the problem of asymptomatic colonization and transmission of *B. pertussis* [224]. The live attenuated vaccine, BPZE1, was specifically developed to be administered by the i.n. route and animal studies have shown that it can generate protective T_RM_ cells that produce IL-17 and IFN-γ, as well as local sIgA in the respiratory tract, and can confer durable protection against nasal colonization with *B. pertussis* [34,92,121,123]. The first-in-human trials showed that the i.n. administration of BPZE1 is safe [122], and it is now being assessed in Phase 2 clinical trials (NCT03541499; NCT03942406).

A pertussis OMV vaccine delivered by the i.n. route generated superior protection and stronger Th1/Th17 response compared with parenteral administration of the same vaccine [137]. Delivery of aP vaccines by the i.n. route also has the capacity to enhance the efficacy of aP vaccines, especially when formulated with adjuvants that stimulate local immunity in the respiratory tract. We have reported that i.n. administration of a three-component aP vaccine, adjuvanted combined with the potent T cell-inducing adjuvant LP-GMP promoted induction of IL-17^+^ T_RM_ cells in the lungs and nasal tissue and conferred long-lived protection against nasal colonization as well as lung infection [33].

## 6. Concluding Remarks

Great strides have been made in recent years on our understanding of the mechanisms of protective immunity against *B. pertussis*, which will assist in the design of next generation pertussis vaccines. Most people in the field now accept that the current alum-adjuvanted patenterally-delivered aP vaccine is suboptimal and needs to be supplemented or replaced with a more effective pertussis vaccine. The discovery from baboon and mouse models that current aP vaccines do not generate protective immunity against nasal colonization has been a game-changer. Together with epidemiological studies in humans, the findings suggest asymptomatic transmission is occurring in vaccinated populations. The demonstration that the current aP vaccines fail to generate CD4 T_RM_ cells in the lung and nasal tissue, alongside the well-established fact that they do not induce mucosal IgA, has focused attention on the need for new vaccine approaches that induce local T cells and antibodies in the respiratory tract. There is strong evidence that IFN-γ-secreting Th1 cells play a key role in clearance of a primary infection and preventing re-infection of the lungs with *B. pertussis*. In contrast, protection in the nasal cavity appears to be more dependent on IL-17 produced by Th17 and possibly also from other sources, including γδ T cells.

The induction of potent Th1- and Th17-type CD4 T_RM_ cells is likely to be a key factor in the design of more effective vaccines. The use of novel adjuvants and vaccine delivery systems in aP vaccines, as well as the development of live attenuated and OMV vaccines for pertussis, are already showing promising results in animal models and more limited early clinical trials. The implementation of new vaccines will not be straightforward, as unbundling of the current pediatric combination would be logistically difficult. The first steps may see the implementation of new booster vaccines involving the existing aP vaccine antigens but with a novel adjuvant. However, emerging data suggest that it may not be easy to shift the Th2 responses set following immunization with current alum-adjuvanted aP vaccine. Therefore, an alternative approach may be to introduce a priming dose with stand-alone pertussis vaccine formulation that induces the desired cellular immune responses in neonates. If *B. pertussis*-specific Th1 and Th17 responses are set early in life prior to immunization with routine pediatric vaccine (including pertussis) formulated with alum, this may be the road to a more effective vaccine that will induce sterilizing immunity in the nasal cavity as well as the lungs. However, a longer term objective, which is likely to be more effective, should be to remove the aP vaccine from the current pediatric combination and replace it with an easily administered nasally-delivered pertussis vaccine, which promotes respiratory T_RM_ cells that confer sustained protective immunity in the nasal mucosae as well as the lungs.
